# Comparative Analysis of Antibody Titers against the Spike Protein of SARS-CoV-2 Variants in Infected Patient Cohorts and Diverse Vaccination Regimes

**DOI:** 10.3390/ijms232012231

**Published:** 2022-10-13

**Authors:** Alexandru Odainic, Jasper Spitzer, Jennifer Barbara Szlapa, Simon Schade, Tim Jonas Krämer, Jakob Neuberger, Christian Bode, Folkert Steinhagen, Ricarda Maria Schmithausen, Gero Wilbring, Esther Sib, Nico Tom Mutters, Frederik Rabenschlag, Lisa Kettel, Maike Woznitza, Kathrin van Bremen, Tina Peers, Gez Medinger, Anushka Kudaliyanage, Maike Kreutzenbeck, Ulrike Strube, Joseph M. Johnson, Dawn Mattoon, Andrew J. Ball, Stefan Scory, Richard McGuire, Christian Putensen, Zeinab Abdullah, Catharina Latz, Susanne Viktoria Schmidt

**Affiliations:** 1 Institute of Innate Immunity, University Hospital Bonn, 53127 Bonn, Germany; 2 Department of Microbiology and Immunology, The Peter Doherty Institute for Infection & Immunity, University of Melbourne, Melbourne, VIC 3010, Australia; 3 Institute of Experimental Immunology, University Hospital Bonn, 53127 Bonn, Germany; 4 Department of Anesthesiology and Intensive Care Medicine, University Hospital Bonn, 53127 Bonn, Germany; 5 Institute for Hygiene and Public Health, University Hospital Bonn, 53127 Bonn, Germany; 6 Medical Corps of the German Armed Forces, German Armed Forces Central Hospital, 56072 Koblenz, Germany; 7 Department of Internal Medicine I, University Hospital Bonn, 53127 Bonn, Germany; 8 Clare Park Hospital, Farnham GU10 5XX, UK; 9 Department of Paramedic Science, School of Health and Social Work, University of Hertfordshire, Hatfield AL10 9AB, UK; 10 Quanterix Corporation, Billerica, MA 01821, USA; 11 Meso Scale Diagnostics, Rockville, MD 20850, USA; 12 Dardenne Eye Hospital, 53177 Bonn, Germany

**Keywords:** antibody response, SARS-CoV-2, vaccination, inflammation, Long-COVID, lymphocytes

## Abstract

The presence of neutralizing antibodies against SARS-CoV-2 correlates with protection against infection and severe COVID-19 disease courses. Understanding the dynamics of antibody development against the SARS-CoV-2 virus is important for recommendations on vaccination strategies and on control of the COVID-19 pandemic. This study investigates the dynamics and extent of α-Spike-Ab development by different vaccines manufactured by Johnson & Johnson, AstraZeneca, Pfizer-BioNTech and Moderna. On day 1 after vaccination, we observed a temporal low-grade inflammatory response. α-Spike-Ab titers were reduced after six months of vaccination with mRNA vaccines and increased 14 days after booster vaccinations to a maximum that exceeded titers from mild and critical COVID-19 and Long-COVID patients. Within the group of critical COVID-19 patients, we observed a trend for lower α-Spike-Ab titers in the group of patients who survived COVID-19. This trend accompanied higher numbers of pro-B cells, fewer mature B cells and a higher frequency of T follicular helper cells. Finally, we present data demonstrating that past infection with mild COVID-19 does not lead to long-term increased Ab titers and that even the group of previously infected SARS-CoV-2 patients benefit from a vaccination six months after the infection.

## 1. Introduction

In December 2019, a new coronavirus named SARS-CoV-2 spread from China, leading to a worldwide pandemic [1]. According to the World Health Organization, multiple SARS-CoV-2 variants of interest and concern, among them B1.617.2 (Delta) and the latest B.1.1.529 (Omicron) [2] led to up to 594 million registered cases through August 2022 [3]. Up to the beginning of April 2022, it caused over 6 million deaths by an acute respiratory distress syndrome (ARDS) and associated complications, as all organs are affected by SARS-CoV-2 infection. Viral clearance by the host’s immune system is essential to restrain viral infections and maintain the host’s cellular homeostasis. Cells of the innate immune system recognize viruses via extra- and intracellular PRRs (pattern recognition receptors). These, in turn, activate immune-defensive signaling cascades via inflammasome activation and secretion of inflammatory cytokines such as IL1α, IL6 and IFNγ (summarized by Diamond and Kanneganti [4]). In severe and critical COVID-19 cases with a poor outcome, hyperinflammation is observed. This hyperinflammation is caused by a dysregulated cytokine release from infected cells and/or subsequent activated immune cells (neutrophils, tissue-resident macrophages, peripheral monocytes and T cells) [5]. Therefore, the staple of treatment for hospitalized patients consists of corticosteroids and non-steroidal anti-inflammatory drugs (NSAR), antibodies (Ab) against the IL6 receptor or inhibitors of inflammatory cytokine-driven signaling cascades [6,7,8].

Binding of the Spike (S)-protein—a part of the viral capsid—to the host’s ACE2 expressing cells allows SARS-CoV-2 to enter human cells [9,10]. The innate immune system responds to the SARS-CoV-2 infection with opsonizing and neutralizing antibodies secreted by B cells, which are taught by antigen-specific T cells. IgG and IgM antibodies usually develop roughly two weeks after symptom onset, with 100% of patients achieving seroconversion after 20 days [11]. The generation of neutralizing antibodies in patients with recovered SARS-CoV-2 infection correlated to the presence of CD134^+^CD25^+^ positive circulating follicular T helper cells and class-switched CD19^+^IgD^−^ B cells specific for the S-protein [12]. At the time of publication, the WHO lists over 300 vaccine projects. The intent is to raise specific Ab titers, thereby preventing the viral transmission and reducing the likelihood of severe and critical COVID-19 disease courses. At the beginning of 2020, only one year after the pandemic emerged, two forms of novel vaccines (mRNA- and vector-based) were approved for use in Europe. Initially, one dose of the vector-based vaccine from Johnson & Johnson (COVID-19 Vaccine Janssen^®^; [13]) and two doses of AstraZeneca (Vaxzevria^®^; [14,15]) were considered sufficient to achieve a primary immunization. Subsequently, a booster with an mRNA-based vaccine was recommended [16]. Both vaccines use a recombinant, replication-incompetent vector of the human adenovirus type Ad26 (Johnson & Johnson, JJ) or chimpanzee adenoviral vector ChAdOx1 (AstraZeneca, AZ, Cambridge, UK), which carries the genetic information for the S-protein of SARS-CoV-2. The general recommended vaccination scheme [17,18] for mRNA-based vaccines from Moderna (Spikevax [19]) or Pfizer–BioNTech (Comirnaty [20]) includes two inoculations with a temporal difference of 4 to 8 weeks and a recommended booster after six months. Both vaccines use mRNA transcripts which encode the full-length Spike protein with a transmembrane anchor and an intact S1–S2 cleavage site [21]. The presence of plasmablasts and germinal B-cell responses, including cross-reactive memory B cells, provides a robust humoral immunity against SARS-CoV-2 after vaccination [22].

The herein-delineated study investigates the dynamics of α-Spike-Ab (α-Spike-Ab) titers in SARS-CoV-2-infected patients with different disease outcomes and diverse vaccination cohorts over a time frame of up to one year. We quantified cytokines, markers for neuroinflammation and phenotypes of B and T cell populations longitudinally via flow cytometry. Infection with SARS-CoV-2 induced S-protein specific antibodies within one week. These Ab titers were significantly reduced after six months. Ab titers after vaccination followed a similar time course, independent of the vaccine. Our data support the current recommendation to boost every 6th or 7th month to maintain a sufficient Ab concentration in the peripheral blood. Our study further demonstrates that recovered COVID-19 patients benefit from booster vaccinations.

## 2. Results

### 2.1. A-Spike-Ab Titers Differ with Disease Severity of COVID-19 and Correlate to Elevated Numbers of Class-Switched B Cells and Tfh Cells

#### 2.1.1. Mild Cohort

**Figure 1:**
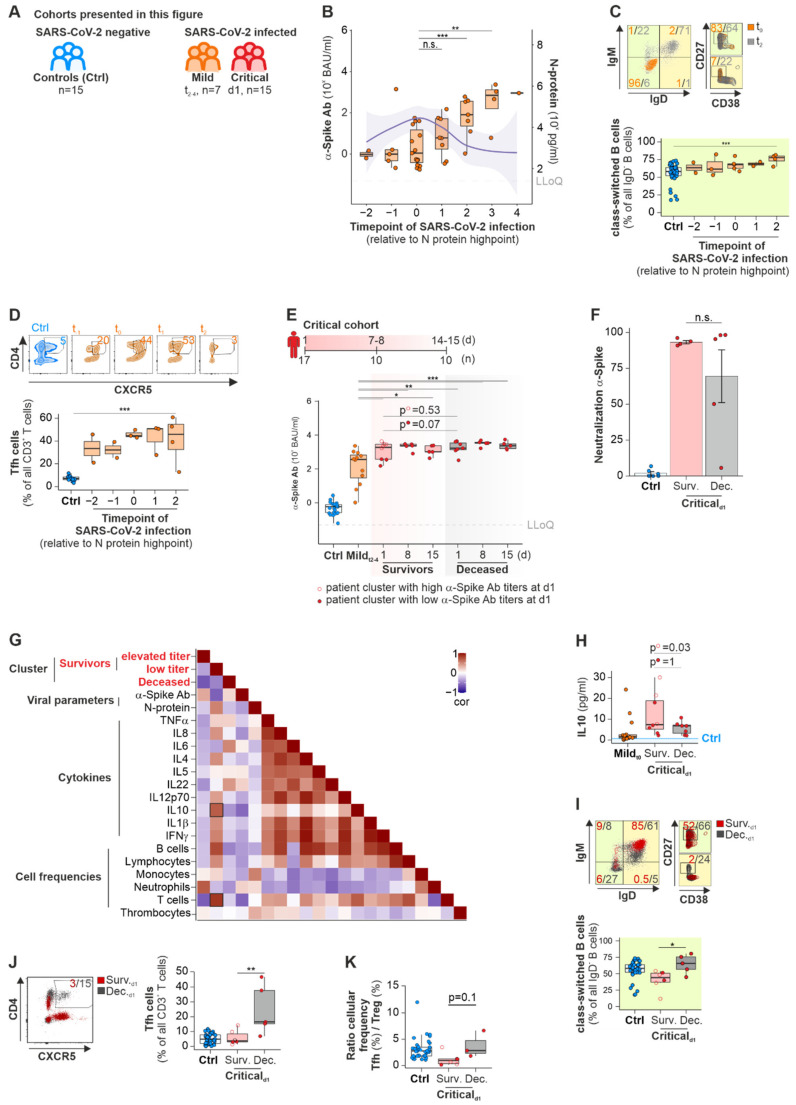
Dynamics of Ab titers against Wuhan SARS-CoV-2 Spike-protein in different COVID-19 disease stages in correlation of B and T cell maturation. (**A**) Scheme of patient cohorts and number of patients. (**B**) Development of α-Spike-Ab titers in SARS-CoV-2 infected patients with a mild disease course harmonized to the timepoint of highest N-protein concentrations (t_0_) in sera. The shaded area indicates the 95% confidence interval of N-protein concentrations. (**C**) Representative dot plot (upper panel) and summarized box plot (lower panel) of B cells (**C**) in patients with mild SARS-CoV-2 disease course. (**D**) Representative contour plots (left panel) and summarized boxplot (right panel) of T follicular helper cells in mildly ill COVID-19 patients. (**E**) Titers of Wuhan α-Spike-Ab concentrations n sera of patients with a critical COVID-19 disease course; non-filled points show the cluster of patients with an increased Ab titer. (**F**) Neutralization capacity of the antibodies against the RBD of Spike protein in healthy non-vaccinated controls and critically ill patients. (**G**) Correlation plot of selected parameters of patients with a critical disease course on day 1 showing Pearson correlation. Bordered squares show significant correlations. (**H**) IL10 titers in sera of patients with critical disease course. (**I**) Flow cytometry data corresponding to (**D**) applied to the critical cohort; non-filled points show cluster of patients with increased Ab titer. (**J**) Representative dot plot (left panel) and quantitative boxplot (right panel) of T follicular helper cells in patients with a critical SARS-CoV-2 course; non-filled points show cluster of patients with increased Ab titer. (**K**) Ratio of the frequencies of T follicular helper cells to T regulatory cells (CD3^+^CD4^+^CD25^high^CD127^−^); non-filled points show cluster of patients with increased Ab titer. Ctrl denotes a cohort of patients with similar symptoms but without a positive SARS-CoV-2 test. Blue line, mean concentration in healthy Ctrl; blue dashed line, 95% confidence interval; LLoQ, Lower Level of Quantification; Mann-Whitney U test, un-adjusted *p*-value (B, C, E, G, H, I, J); * *p*Val < 0.05, ** *p*Val < 0.01, *** *p*Val < 0.001.

We analyzed Ab development in the initial stage of SARS-CoV-2 infection, its persistence throughout hospitalization and the duration of Ab presence over a period of up to 12 months in three independent cohorts (Figure 1A). Patients of similar age (mean_yrs_: Controls = 57, Mild = 54, Critical = 63; Supplementary Figure S1A) and with comparable systemic diseases such as hypertension (21–41%) and diabetes (7–12%) served as controls. We observed equal sex ratios in all cohorts, except for the critically ill patients. Male patients were overrepresented (88%) in this group—a phenomenon described early on during the first year of the SARS-CoV-2 pandemic [23].

We analyzed the development of α-Spike-Ab (Wuhan variant) in the early stages of the infection in 15 patients with mild COVID-19 disease. This group of patients did not need medical attention and recovered from the infection at home. Blood samples were collected at multiple timepoints during the first eight days and on day 15 after enrolment into the study (Figure 1B). As the onset of infection cannot be defined precisely in most cases, we quantified the patients’ highest serum level for SARS-CoV-2 Nucleoprotein (N-protein). The day of the highest concentration of N-protein in serum of SARS-CoV-2-infected patients was set as t_0_ and this enabled us to harmonize the analysis for the complete cohort [24]. The samples collected at preceding timepoints were designated t_-1_ and t_-2_, whereas timepoints following t_0_ were set as t_1–4_. The onset of infection is presumed to be up to 8 days before t_0_. Concentrations of α-Spike-Ab started to increase after one-to-three days (t_1_) of infection. They became significantly upregulated from day seven on after t_0_ (t_2_, Figure 1B). N-protein levels showed an opposite trend, which we interpret as a sign of viral clearance as demonstrated by us before [24]. Titers of α-Spike-Ab against the alpha-, beta- and gamma-variant of SARS-CoV-2 were quantified (Supplementary Figure S1B,C). Low titers of specific α-Spike-Ab against the respective SARS-CoV-2 variants were observed at t_2_ (Supplementary Figure S1B). Moreover, rising α-Spike-Ab titers (Wuhan variant) resulted in increasing differences in variant immunity (t_2_ and t_3_, Supplementary Figure S1C). To enable the comparison between the antibody response against the different virus strains, we plotted their respective concentration reduction when compared to the Wuhan strain in percent: for example, Ab concentrations against the Spike protein of the alpha (17%), beta (51%) or gamma (50%) variant were lower than α-Spike-Ab concentrations against the Wuhan variant at t_2_ (Supplementary Figure S1C).

To investigate the dynamics of B and CD4^+^ T cells contributing to the humoral immune responses in SARS-CoV-2 infected patients, we performed flow cytometry analysis for different B cell subpopulations and T follicular helper cells (Tfh) in the peripheral blood of the different groups. In comparison to the control group, SARS-CoV-2-infected patients showed higher percentages of pro-B cells (mean and SEM for Ctrl: 33 ± 3%; Mild_t0_: 93 ± 1%), especially at t_0_ of infection (Supplementary Figure S1D). The percentage of mature B cells was decreased at t_0_ (mean and SEM for Ctrl: 53 ± 4; Mild_t0_: 3 ± 0%) whereas the percentage of immature B cells increased from t_0_ on (mean and SEM for Mild_t0_: 3 ± 1%; Mild_t2_: 7 ± 2%). Significantly elevated numbers of IgD^−^CD38^low^CD27^+^ class-switched B cells (Ctrl: 58 (52–64)%; Mildt2: 78 (73–80)%; Figure 1C) correlated with increased concentrations of α-Spike-Ab after 7 days (t2) after the onset of infection (t_2_, *p* = 0.01, r = 0.59). In contrast, no significant changes were detected for non-class-switched B cells (IgD^+^CD38^low^CD27^+^) (median, interquartile range for Ctrl: 10 (6–16)%; Mildt2: 9 (9–10)%; Supplementary Figure S1E). Furthermore, percentages of CD4^+^CXCR5^+^CD3^+^ Tfh cells, required for the differentiation of the Ab production by plasma cells, were elevated in the SARS-CoV-2 infection in comparison to numbers detected in uninfected controls (median, interquartile range for Ctrl: 7 (6–9)%; Mild_t-2-t2_: 32–51%; Figure S1D).

#### 2.1.2. Critical Cohort

To investigate whether α-Spike-Ab concentrations between mild and critical COVID-19 patients differ and hypothetically could predict the severe forms of COVID-19, titers of α-Spike-Ab were monitored from admission to the ICU until discharge from the hospital, death, or at least up to 15 days of hospitalization (Figure 1E). There was a significant difference in α-Spike-Ab concentrations between mild and critically infected COVID-19 patients who died from SARS-CoV-2 infection (*p*_d1_ = 0.005, *p*_d8_ = 0.0006, *p*_d15_ = 0.001, Figure 1E) and those patients who survived COVID-19 (*p*_d1_ = 0.04, *p*_d8_ = 0.004, *p*_d15_ = 0.03, Figure 1E). Similar to patients with mild forms of COVID-19, Ab titers for the alpha-, beta- and gamma variant of SARS-CoV-2 in patients with critical SARS-CoV-2 infection were reduced in comparison to α-Spike-Ab against the original Wuhan variant (Supplementary Figure S1F). Comparing the α-Spike-Ab concentrations between survivors and deceased patients, we did not find any significant differences over the duration of study (Figure 1E). Following the hypothesis that severe disease courses with poor outcome might be linked to low affinity of IgG antibodies against the Receptor Binding Domain of the Spike protein of SARS-CoV-2, we performed neutralization assays against the initial strain as well as alpha, beta and gamma variants of concern. In this assay, neutralizing antibodies in the sera of COVID-19 patients compete with human ACE2 protein standard for binding to SARS-CoV-2 RBD antigens (Wuhan, alpha, beta and gamma strains) immobilized on the plate surface. In contrast to the neglectable presence of neutralizing antibodies in the control group (mean and SEM for Ctrl Wuhan: 2 ± 1%; alpha: 3 ± 1%; beta: 3 ± 2%; gamma: 10 ± 2%), specific neutralizing antibodies were found in the group of severe COVID-19 patients with variable neutralizing capacity against four SARS-CoV-2 variants (Figure 1F, Supplementary Figure S1G). The neutralizing capacities varied not only between the virus strains, but also between the survivor and deceased patients, yet these differences were found to be not significant (mean and SEM for Wuhan Surv.: 93 ± 1%, Dec.: 70 ± 18%; alpha Surv.: 86 ± 3%, Dec.: 65 ± 18%; beta Surv.: 45 ± 6%, Dec.: 36 ± 15%; gamma Surv.: 59 ± 6%, Dec.: 47 ± 14%). Independent of the disease outcome, the capacity to neutralize SARS-CoV-2 Spike-RBD of the beta variant was the lowest among the analyzed strains (Supplementary Figure S1G). As observed by Garcia-Beltran et al. [25], the titers of α-Spike-RBD-Ab correlated positively to its percent of neutralization capacity of RBD-specific antibodies against the four tested SARS-CoV-2 variants (Supplementary Figure S1H). For the survivor group, we observed a separation into either high or low α-Spike-Ab titers at d1 of admission to the ICU ward. This was independent of the viral load, as we did not observe any differences in the N-protein concentrations in these patients’ sera. In contrast, all patients in the deceased group showed high α-Spike-Ab throughout hospitalization. By Pearson correlation analysis, we integrated additional data on cytokine concentrations and blood immune cell frequencies of critically ill patients to investigate if the low α-Spike-Ab titers of COVID-19 survivors correlated to deviations in the immune response. The anti-inflammatory cytokine IL10 and the T cell counts correlate positively to low α-Spike-Ab titers of COVID-19 survivors (Figure 1G,H). Not distinguishing patient clusters, critically ill patients who survived the SARS-CoV-2 infection showed a trend to higher IL10 concentrations (*p* = 0.28, Figure 1H). This tendency for higher IL10 serum concentrations became significant when distinguishing the group of survivors by the α-Spike-Ab titer: Higher α-Spike-Ab titers correlated positively with high IL10 concentrations. Critically ill COVID-19 patients who died from SARS-CoV-2 infection showed a decreased frequency of mature B cells in the blood on the day of admission to the ICU (Supplementary Figure S1I), but higher frequencies of pro B cells (mean and SEM for Ctrl: 33 ± 3%; Surv.: 17 ± 3%; Dec.: 58 ± 12%). The percentage of immature B cells was lower in the uninfected controls compared to deceased or survivors with similar rates (mean and SEM for Ctrl: 3 ± 0%; Surv.: 15 ± 6%; Dec.: 10 ± 4%). Of note, deceased patients showed higher percentages of class-switched memory B cells compared to those who survived the infection (median, interquartile range for Surv.: 44 (37–51)%; Dec.: 66 (57–76%; Figure 1I). Yet, no significant differences were found for class-switched memory B cell frequencies between survivors with high and low α-Spike-Ab titers (*p* = 0.8). Similar observations were made for Tfh cells (median, interquartile range for Surv.: 4 (3–10)%; Dec.: 17 (15–38)%; Figure 1J). In contrast, no significant differences in percentages of non-class switched B cells were observed (median, interquartile range for Surv.: 8 (5–12)%; Dec.: 8 (4–24)%; Supplementary Figure S1J). The ratio of Tfh and Treg cell frequencies indicates an ongoing inflammatory immune response and autoimmune diseases [26]. We observed that patients with a critical disease course who died from the infection had a 3.2-fold elevated ratio of Tfh/Treg cells compared to those who survived (Figure 1K). This observation was independent of the differences of α-Spike-Ab titers found in the group of survivors, which we interpret as part of a dysregulated immune response fostered on different levels, like lack of anti-inflammatory cytokines, like IL10 and dysregulation of Treg cells. Previously, a low ratio between IFNγ and IL10 has been associated with viral infection [27]. Calculating the IFNγ/IL10 ratio for critical patients, we did not observe any differences between non-infected control participants and critical patients who survived or died from SARS-CoV-2 infection. Therefore, we conclude that the usage of the IFNγ/IL10 ratio as a predictive marker for life-threatening SARS-CoV-2 outcomes is not adequate.

#### 2.1.3. Long COVID

**Figure 2:**
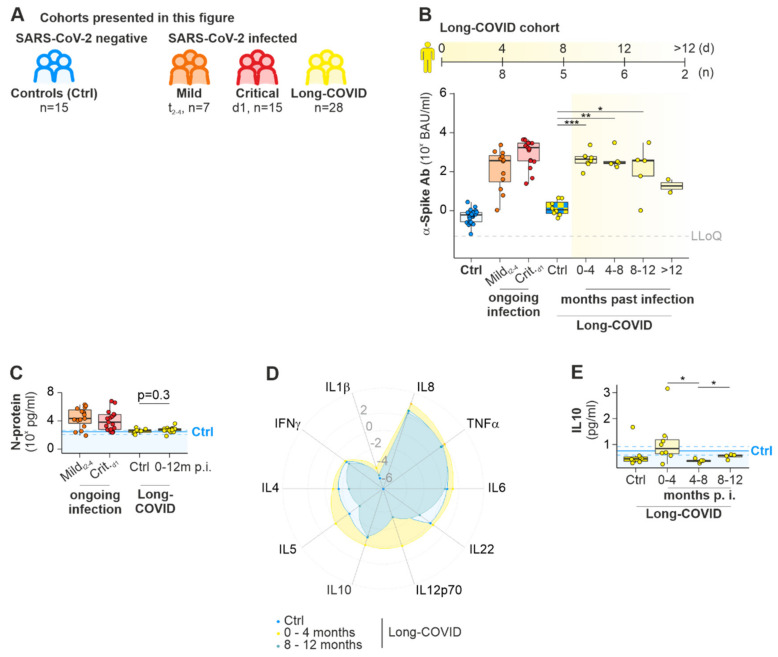
Dynamics of Ab titers against Wuhan SARS-CoV-2 Spike-protein and cytokines in Long-COVID. (**A**) Scheme of patient cohorts presented in the figure. (**B**) Comparison of α-Spike-Ab titers of included Long-COVID volunteers after various timepoints of infection to healthy controls, patients with a mild and critical COVID-19 disease course, as well as Long-COVID controls. (**B**–**E**) Characterization of the Long-COVID cohort by α-Wuhan-Spike-Ab (**B**), N-protein (**C**), normalized cytokine titers in a radar plot (**D**), and IL10 titers (**E**). Ctrl denotes a cohort of patients with similar symptoms but without a positive SARS-CoV-2 test. Blue line, mean concentration in healthy Ctrl; blue dashed line, 95% confidence interval; LLoQ, Lower Level of Quantification; Mann-Whitney U test; * *p*Val < 0.05, ** *p*Val < 0.01, *** *p*Val < 0.001.

Neurath et al. [28] hypothesized a persistence of SARS-CoV-2 in unknown reservoirs after an acute, cured infection, causing an ongoing form of low-grade inflammation and/or neuronal damage. We asked: Does an ongoing immune response, as seen by a continuous production of antibodies against the Spike protein and production of cytokines by immune cells, could contribute to the development of Long-COVID? Our cohort of 20 Long-COVID patients suffered from long-term complications such as fatigue, tachycardia or high blood pressure (summarized under “cardiovascular system”), headaches and insomnia. (Figure 2A, Supplementary Figure S2A,B). We age-matched these Long-COVID patients with patients suffering from comparable symptoms (LC control, mean_yrs_: 40; Long-COVID, mean_yrs_ = 39; Supplementary Figure S2A,B). Ab titers against Spike protein of different SARS-CoV-2 variants in Long-COVID patients were elevated up to eight months after infection and decreased over the observation period of 12 months (Figure 2B, Supplementary Figure S2C). In contrast, α-Spike-Ab concentrations in sera of LC controls were reduced 392-fold compared to Long-COVID_0–4 m_ (median, interquartile range for LC controls: 1 (1–39) BAU/mL; Long-COVID_0–4 m_: 444 (278–600) BAU/mL) and did not differ in α-Spike-Ab titers to healthy-aged matched controls (median, interquartile range for Ctrl: 1 (0–1) BAU/mL), which further supports the lack of infection with SARS-CoV-2 in this control group (Figure 2B). Despite elevated α-Spike-Ab titers, we did not detect any SARS-CoV-2 N-protein in LC patients (means for Long-COVID_0–12 m_: 594 pg/mL; LC control: 477 pg/mL; Figure 2C) in comparison to acutely infected patients (means for Mild_t2–4_: 1072 pg/mL; Critical_d1_: 6927 pg/mL). We tested the serum samples for classical inflammatory and anti-inflammatory cytokines as systemic markers for a prolonged immune response, and observed a trend for slightly elevated cytokine levels for IL8, TNFα, IL6, IL12p70, IL10, IL5 and IL4 in the first four months after the infection (Figure 2D). IL12p70 was significantly elevated in Long-COVID patients up to 4 months after infection (means for LC control: 0.00 pg/mL; Long-COVID_0–4 m_: 0.13 pg/mL; Supplementary Figure S2D). Four months after the infection and later, we found significantly increased IL10 concentrations (means for Long-COVID_0–4 m_: 0.84 pg/mL; Long-COVID_4–8 m_: 0.38 pg/mL; Figure 2E). Further, we tested the serum for markers of neuroinflammation and -degeneration. There were no significant differences in Aβ40, Aβ42, GFAP and NF-light concentrations between the LC and LC controls (Supplementary Figure S2E). Therefore, we could not correlate neuroinflammation and -degeneration to Long-COVID symptoms.

In summary, we demonstrate that class-switched memory B cells increase from day seven after infection together with rising α-Spike-Ab titers. We observed that patients with mild symptoms, critically ill patients as well as Long-COVD patients, display comparable levels of α-Spike-Ab titers. Critically ill patients who survived COVID-19 and Long-COVID patients showed elevated levels of the anti-inflammatory cytokine IL10, which was independent of N-protein levels. In contrast, patients who died from SARS-CoV-2 infection showed reduced IL10 levels as well as a higher percentage of class-switched memory B cells and Tfh cells. The ratio for the cellular frequency of Tfh/Treg was elevated. This could indicate the induction of an autoimmune response by SARS-CoV-2 in those critically ill patients who died from the disease.

### 2.2. mRNA-Based COVID-19 Vaccines Lead to a Temporal Immunity against SARS-CoV-2

**Figure 3:**
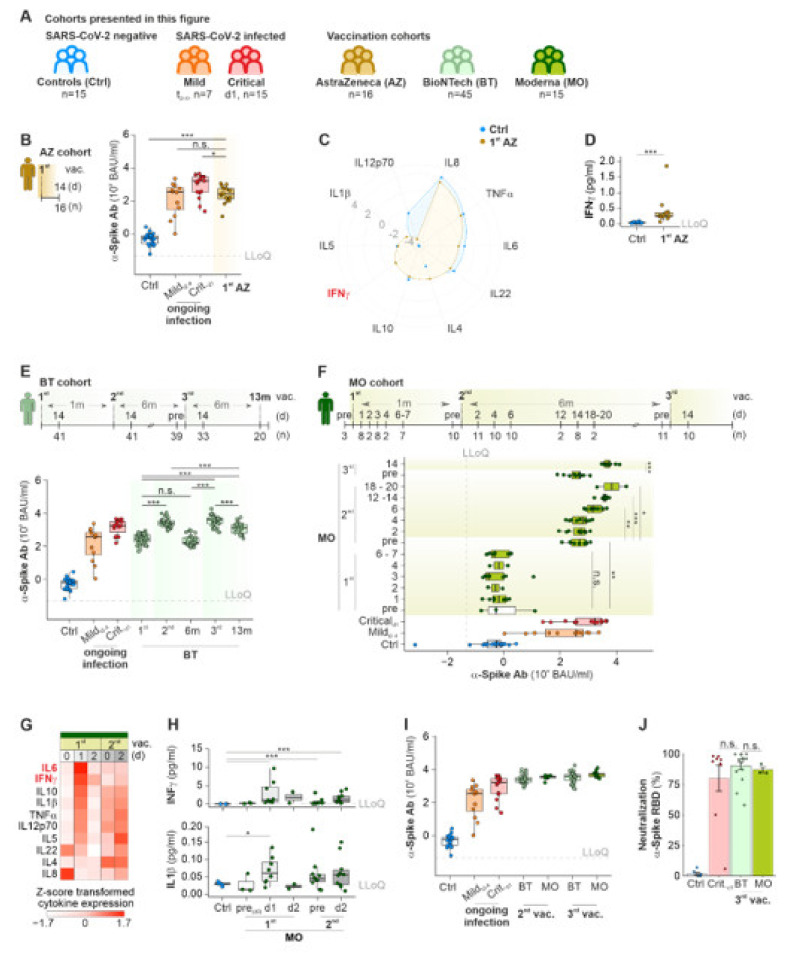
Dynamics of Ab titers against Wuhan SARS-CoV-2 Spike-protein in different vaccination regimens. (**A**) Scheme of vaccination cohorts and groups of SARS-CoV-2 infected patients. (**B**–**D**) Concentrations of α-Spike-Ab (**B**), cytokines (**C**) including IFNϒ (**D**) in sera of participants 14 d after vaccination with AZ. (**E**,**F**) Scheme of vaccination strategy and longitudinal analysis of α-Spike-Ab titers in participants multiple times vaccinated with BT (**E**) or MO (**F**). (**G**) Heatmap of Z-score transformed cytokine concentrations in blood of participants vaccinated with MO before and shortly after the vaccination. (**H**) Cytokine concentrations for IFNϒ (top) and IL1β (bottom) before and after vaccination with MO. (**I**) Comparative analysis for α-Spike-Ab titers after multiple inoculations with mRNA vaccines. (**J**) Neutralization capacity of antibodies against α-Spike-RBD of SARS-CoV-2 in healthy non-vaccinated controls, critically ill COVID-19 patients, and vaccinated individuals with BT and MO six months after the second booster. LLoQ, Lower Level of Quantification; Mann–Whitney U test, un-adjusted *p*-value (A), adjusted *p*-value (B, E); * *p*Val < 0.05, ** *p*Val < 0.01, *** *p*Val < 0.001.

From December 2020 on, three COVID-19 vaccines were available in Europe: the vector-based vaccine ChAdOx1nCOV-19 by AstraZeneca (AZ), and two mRNA-based vaccines by Pfizer–BioNTech (BT) and Moderna (MO). We recruited 76 volunteers for three representative cohorts with slightly different vaccination strategies (Figure 3A, Supplementary Figure S3A) to study the timing and amount of α-Spike-Ab developed in response to each vaccination. After the first inoculation with AZ, titers of α-Spike-Ab against the Wuhan and to a lesser extent alpha-, beta- and gamma SARS-CoV-2 variants were increased and comparable to levels of patients with an ongoing SARS-CoV-2 infection (Figure 3B, Supplementary Figure S3B). Participants reported adverse events like, fever, chills, head and body aches. These flu-like symptoms are hypothetically caused by an activation of the immune system, leading to the production of pro- and anti-inflammatory cytokines. We quantified 10 classical cytokines participating in immune responses in the blood of AZ vaccinated participants 14 days after the first inoculation. IFNγ was the only investigated cytokine that increased significantly with AZ (means for Ctrl: 0.04 pg/mL; 1st AZ: 0.44 pg/mL; Figure 3C,D). IFNγ is released by CD4^+^ T_H_1 cells. Its serum levels can serve as an indicator for the induction of a broad T-cell response against the S antigen, as observed before for other replication-deficient adenoviral vectors [29].

An initial vaccination with Comirnaty from Pfizer–BioNTech induced α-Spike-Ab titers in healthy participants comparable to COVID-19 patients with ongoing SARS-CoV-2 infection at early time points (medians for Mild_t2–4_: 369 BAU/mL; 1st BT: 272 BAU/mL; Figure 3E). Similar to the AZ-vaccination and active ongoing SARS-CoV-2 infections, α-Spike-Ab titers against SARS-CoV-2 variants were reduced by up to 50% (Supplementary Figure S3C). The second vaccination with BT led to a significant booster effect with increased concentrations of α-Spike-Ab (median for 2nd BT: 2488 BAU/mL); albeit, the booster effect did not last very long: 6 months after the booster vaccination, α-Spike-Ab titers were reduced to levels lower than 2 weeks after the initial vaccination (median for BT_6m_: 164 BAU/mL). A third vaccination was able to restore and moreover increase the α-Spike-Ab titers even beyond the concentrations achieved 14 days after the second vaccination with BT (median for 3^rd^ BT: 3920 BAU/mL). Six months after the third vaccination, titers for α-Spike-Ab returned to lower yet potent levels (median for BT^13m^: 1283 BAU/mL).

We observed similar dynamics of α-Spike-Ab titers for mRNA-1273 (Spikevax^®^) from Moderna (MO). Six days after the initial vaccination with MO, α-Spike-Abs were not yet detectable (median for MO_d6–7_: 0 BAU/mL; Figure 3F). Yet, after four weeks, titers of α-Spike-Abs against the Wuhan variant of SARS-CoV-2 and to a lesser extent to other variants (Supplementary Figure S3D) were increased. Six to 20 days after the booster, titers increased rapidly (medians for 2nd MO_pre_: 483 BAU/mL; 2nd MO_d18–20_: 12,221 BAU/mL). Comparable to BT, α-Spike-Ab titers were reduced to basal immunization titers 6 months after the booster (median for 3rd MO_pre_: 484 BAU/mL). Yet, a third vaccination enabled the α-Spike-Ab titers to be elevated again to levels comparable to the titers after the second vaccination (median for 3rd MO_d14_: 5001 BAU/mL). Participants vaccinated with MO reported flu-like symptoms, similar to volunteers vaccinated with AZ. To investigate, if vaccination with MO leads to an inflammatory response, the presence of pro- and anti-inflammatory cytokines was quantified in the sera of participants before, on days one and two after the first inoculation, and before and two days after the second vaccination with MO (Figure 3G). Inflammatory cytokines like IL6, IFNγ, IL1β and TNFα were induced 24 hrs after the first inoculation (Supplementary Figure S3E), of which only IFNγ (medians for Ctrl: 0.03 pg/mL; 1st MO_d1_: 1.16 pg/mL) and IL1β (medians for Ctrl: 0.03 pg/mL; 1st MO_d1_: 0.06 pg/mL) were significantly upregulated by the initial vaccination (Figure 3H). This pro-inflammatory immune response was short-lived as the concentrations of pro-inflammatory cytokines were reduced to control levels at d2 after vaccination. We did not detect any significant increase in cytokine levels following the second inoculation in comparison to levels before the initial vaccination (pre_d0_).

There was neither significant difference between Ab titers induced by BT or MO components 14 days after the second and third vaccination nor between the test regimes at the chosen time point (Figure 3I). We completed our analysis on the efficiency of COVID-19 vaccines by studying the neutralization capacity of α-Spike-Abs induced by BT or MO vaccines, six months after the second booster vaccination (3rd vac). Antibodies in the serum of vaccinated participants showed similar neutralization of Spike RBD of the Wuhan and alpha variant to critically ill COVID-19 patients (Figure 3J, Supplementary Figure S3F). Despite the reduced number of data points for the MO test cohort, we observed a significant stronger neutralization against the Spike RBD of beta and gamma SARS-CoV-2 strains by the antibodies developed after BT vaccination (Supplementary Figure S3F).

It can be concluded that both mRNA-based vaccines show similar efficiencies in the induction of Ab titers against SARS-CoV-2 variants. Additionally, we observed decreasing Ab titers against the Spike protein over a period of six months after the inoculation for all herein-investigated mRNA vaccines.

### 2.3. Cross-Vaccination with Different COVID-19 Vaccines Does Not Exceed Ab Titers against Spike-Protein Achieved with Mono-Vaccine Usage

**Figure 4:**
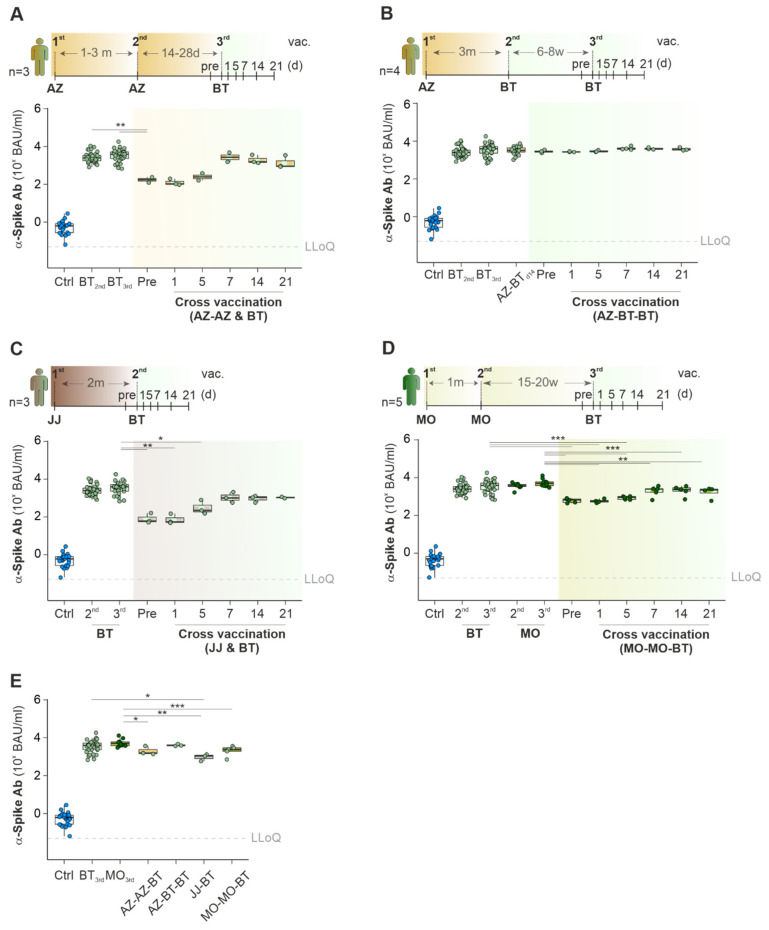
Comparative analysis of Ab titers against Wuhan SARS-CoV-2 Spike-protein in vaccination regimens with multiple vaccines. (**A**–**E**) Vaccination scheme (top) and concentrations of α-Spike-Ab over time (bottom) in cohorts vaccinated with various combinations (as indicated by the scheme) of AZ-AZ-BT (**A**), AZ-BT-BT (**B**), JJ-BT (**C**), and MO-MO-BT (**D**). (**E**) Comparison between the various vaccination schemes; LLoQ, Lower Level of Quantification; Man–Whitney U test, unadjusted *p*-value (**A**), adjusted *p*-value (**C**–**E**); * *p*Val < 0.05, ** *p*Val < 0.01, *** *p*Val < 0.001.

Due to vaccine shortage and adverse events, including cerebral vein thrombosis after COVID-19 vaccination (summarized by Jaiswal et al. [30]), recommendations on target groups for AZ, JJ, BT or MO were revised. At the time of writing this manuscript (August 2022), the German council for vaccines (STIKO, Robert Koch Institute (RKI), Berlin, Germany) recommended combining AZ as a first inoculation with mRNA-based COVID-19 vaccines as a booster or two vaccinations with AZ for citizens over the age of 60 [31]. In total, 3 healthy participants initially vaccinated twice with AZ followed by one inoculation with BT were included in the study (Figure 4A, Supplementary Figure S4A). As established before, a single vaccination with AZ led to a significant increase of α-Spike-Ab concentrations against the original Wuhan strain but also to other SARS-CoV-2 variants (Figure 3B, Supplementary Figure S2B). A booster vaccination with BT up to 28 days after the 2nd AZ lead to a 13-fold increase in levels of α-Spike-Ab from d7 (medians for BT_pre_: 181 BAU/mL; BT_d7_: 3153 BAU/mL; Figure 4A). These levels were comparable to those of BT controls after two inoculations. Additionally, we investigated the α-Spike-Ab titers of four participants whose vaccination schemes followed a different recommendation of the German Council for vaccines (Figure 4B). These participants were inoculated once with AZ and twice with BT. Their α-Spike-Ab titers were compared to controls of participants who received in total three inoculations with BT. Due to the short time between the first and second BT vaccination (<8 weeks), α-Spike-Ab concentrations before the second BT vaccination were already elevated (median for 2nd BT_pre_: 2781 BAU/mL) to comparable levels of participants who received two or three inoculations with BT only (medians for BT_2nd_: 2488 BAU/mL; BT_3rd_: 3920 BAU/mL). No further increase of α-Spike-Ab concentrations was observed up to 21 d after the second BT vaccination (^2nd^ BT_d21_: 3468 BAU/mL).

In contrast to the test group which followed the vaccination scheme AZ-BT-BT, three participants received only one inoculation with JJ. They showed significantly lower α-Spike-Ab titers after two months (median for JJ_pre_: 61 BAU/mL; Figure 4C). One additional vaccination with BT increased the titers significantly to 1007 BAU/mL after seven days. These values were comparable to the BT controls (medians for BT_2nd_: 2488 BAU/mL; BT_3rd_: 3920 BAU/mL). In conclusion, participants vaccinated with a single JJ vaccination benefit from a booster vaccination with BT.

Finally, we observed that participants, who were initially vaccinated two times with MO and switched for the third vaccine to BT, did not benefit from this vaccination strategy (Figure 4D): their α-Spike-Ab titers were two-fold lower in comparison to participants who received three vaccinations with MO (medians for BT_d14_: 2347 BAU/mL; MO_3rd_: 5001 BAU/mL).

In summary, all investigated diverse vaccination strategies resulted in satisfactory α-Spike-Ab titers against Wuhan and other variants 14 days after the last booster (Figure 4E, Supplementary Figure S4B). Yet, we observed significant lower α-Spike-Ab titers between cohorts with a mixed vaccination scheme than the respective BT_3rd_ or MO_3rd_ controls.

### 2.4. Cured COVID-19 Patients Benefit from COVID-19 Vaccination from 6 Months on after the Infection

**Figure 5:**
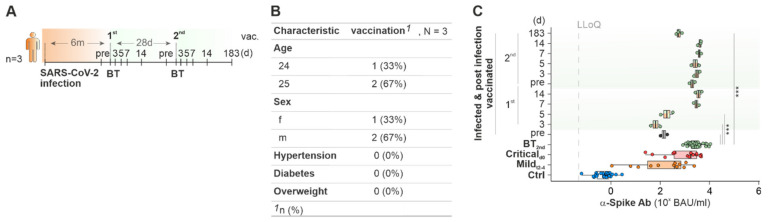
Ab titers against Wuhan SARS-CoV-2 Spike protein after infection and subsequent vaccination over time. (**A**) Vaccination scheme, (**B**) table of clinical characteristics and (**C**) Concentrations of α-Spike-Ab of patients after infection and subsequent vaccination at indicated timepoints. LLoQ, Lower Level of Quantification; Mann–Whitney U test; unadjusted *p*-value (**C**); *** *p*Val < 0.001.

Infection with SARS-CoV-2 mounted a rapid generation of antibodies against the Spike protein during the initial phase of the immune response within the first seven days (Figure 1B). Data from Long-COVID patients showed that α-Spike-Ab titers started to diminish after eight months (Figure 1J). Moreover, they continued to decrease over a period of up to 12 months. To protect against re-infection with SARS-CoV-2, three patients with cured COVID-19 were inoculated twice with BT six months after SARS-CoV-2 infection (Figure 5A). These patients were categorized as healthy as they did not have any diagnosis of hypertension or diabetes, nor were they overweight (Figure 5B). Titers of α-Spike-Ab in sera of cured COVID-19 patients were significantly lower (median for 1st BT_pre_: 148 BAU/mL; Figure 5C) 6 months after infection compared to titers of healthy volunteers vaccinated twice with BT (median for BT_2nd_: 3488 BAU/mL). Interestingly, cured COVID-19 patients showed higher relative α-Spike-Ab titers against the alpha- and gamma-variant, similarly to patients with acute SARS-CoV-2 infection when contrasted to vaccinated participants (Supplementary Figures S1C,F, S3C,D and S5A). Inoculation of cured COVID-19 patients with one dose of BT led to a strong elevation of α-Spike-Ab concentrations in sera after seven days (median for BT_7d_: 3008 BAU/mL) which were comparable to BT controls. A second vaccination after one month with BT did not increase the α-Spike-Ab titers beyond concentrations achieved by the first BT dose (median for 2nd BT_d7_: 3918 BAU/mL). It is worth emphasizing that infection with SARS-CoV-2 and additional vaccination with BT did not lead to a long-lasting Ab production against the Spike protein: 183 days after the vaccination, α-Spike-Ab had decreased to a basal level of 544 BAU/mL (Figure 3F). Overall, our data support the current scientific opinion that even cured COVID-19 patients benefit from an mRNA-based COVID-19 vaccine six months after infection [32,33]. A single SARS-CoV-2 infection does not lead to longer-lasting nor higher levels of Ab titers than vaccination.

## 3. Discussion

At the beginning of the SARS-CoV-2 pandemic there was an urgent need to prevent high incidences of critically ill COVID-19 patients because health care systems around the globe were flooded by intensive-care units requiring COVID-19 patients. The situation was dramatically worsened when medical health care workers became infected and had to temporarily resign from their duties. Intensive efforts to develop effective vaccine against SARS-CoV-2 were initiated, with the aim of raising a strong humoral immune response through the production of neutralizing antibodies against the Spike protein of SARS-CoV-2. On March 18 2020, the U.S Food and Drug Administration (FDA) and the European Medicines Agency (EMA) jointly chaired the first global regulators meeting to discuss regulatory strategies to facilitate the development of SARS-CoV-2 vaccines [34]. Through the US-funded Operation Warp Speed initiative, phases (phase 1–3) of a clinical approval study normally conducted sequentially were instead conducted in parallel. On December 10, 2020, the safety and efficacy of the BNT162b2 mRNA COVID-19 Vaccine were published [20]. On December 11, 2020, the FDA issued the first emergency use authorization (EUA) for BNT162b2 for the mitigation of COVID-19 caused by SARS-CoV-2 in individuals 16 years of age and older [35]. Vaccines by MO, AZ and JJ followed in this order, so that at the end of 2020, just one year after the initial outbreak, several vaccines were licensed for usage in the US and Europe. The approval studies had a mean follow up of only two months, leaving a number of unexplored questions about Ab dynamics. A number of subsequent studies have compared different vaccines [13,14,20,36,37]. Secondary to the generation of neutralizing antibodies against the Spike-protein [38], vaccination led to lower viral loads in previously vaccinated COVID-19 patients [39].

### 3.1. Antibodies

Obesity and related co-morbidities have been reported to correlate with negative disease outcomes [40,41]. We investigated how this correlation translates to antibody titers against SARS-CoV-2 in our study, but we were not able to find any significant correlations in our patient cohorts with regards to obesity, hypertension or diabetes and anti-spike antibody titters. Independent of the vaccine used or the disease stage of COVID-19, we observed a certain range of maximum IgG α-Spike-Ab titers generated (Figure 3, Figure 4 and Figure 5), and these titers were comparable to patients vaccinated once with any kind of COVID-19 vaccine (Figure 3B,E,F). Similar results were found by Brochot et al. [42], who showed that Ab titers against Spike- and N-protein reached a plateau after 14 days of infection onset. Others observed a similar dynamic and plateau for IgM antibodies against the Spike-protein [36]. Due to the half-life of 184 days of α-Spike-Abs [43], a reduction of Ab titers was expected. Indeed, we observed that α-Spike-Abs titers were reduced in cured COVID-19 and Long-COVID patients 6 months after the infection (Figure 1K and Figure 5C). A similar observation was made in a cohort study that focused on the long-term health consequences of COVID-19 patients discharged from the hospital [44]. Post-infection vaccination with mRNA vaccines increased the Ab titers to those of the vaccine control groups (Figure 5C). These results suggest that even cured COVID-19 patients benefited from a mRNA vaccination 6 months after the infection, consistent with CDC, the recommendation for vaccination of all former COVID-19 patients several months after the infection [32,33]. We observed that three vaccine inoculations of BT led to the production of long-lasting (>6 months) α-Spike-RBD-Abs with high neutralizing potential, not only for the initial Wuhan SARS-CoV-2 strain but also against other variants of concern (alpha, beta and gamma; Supplementary Figure S3F). Moreover, we show that beta-specific antibody titers and their neutralizing capacity in vaccinated individuals (Supplementary Figure S3F) and severe COVID-19 patients (Supplementary Figure S1G) were lower than for the Wuhan, alpha and gamma strains. Similar observations were made in nonhuman primates. The beta variant was the most neutralization- and vaccine-resistant of the first four variants of concern, including the delta strain [45]. In contrast, Newman et al. observed no neutralizing capacity against the beta variant of SARS-CoV-2 in sera of elderly people vaccinated twice with BT [46]. The differences in neutralization potential of α-Spike-Ab between the studies might be explained on the one hand by the presence of younger participants in our study, and on the other, by a third inoculation with the mRNA vaccine after six months. We conclude from these results that a third vaccination or inoculation with adapted vaccines against newer variants might be necessary for a robust antibody response against SARS-CoV-2 variants of concern.

### 3.2. Cytokines

Despite the fact that the vast majority of COVID-19 vaccines were well tolerated, a minority of individuals who received a vaccination reported side effects, such as redness and swelling at the side of injection. Mild side effects included flu-like symptoms [47], but serious side effects were also reported, including myocarditis (reviewed by Ling et al. [48]) and thrombosis [49]. Of note, none of the participants in this study reported any serious side effects. The participants in our study reported common and mild side effects like pain at the site of vaccination, headache or fatigue, and in rare cases fever, which were temporary and disappeared after one day. Severe hyper-inflammatory reactions are rare but have also been reported after inoculation with mRNA and DNA vaccines against COVID-19 [50]. As abnormal cytokine concentrations are the driving factor behind hyper-inflammatory reactions, Arunchalam et al. [51] investigated cytokine titers following BT vaccination and found that especially IFNγ was increased after the second inoculation with BT. We can confirm this observation also for vaccinations with AZ (Figure 3A) and MO (Figure 3G,H). Moreover, we observed in participants’ sera vaccinated with MO an increase in the highly pro-inflammatory cytokine IL1β 24 h after the inoculation (Figure 3G,H). Cytokine concentrations were reduced back to normal ranges two days after the inoculation, suggesting that mRNA- and vector-based COVID-19 vaccines cause a temporal, short-lived inflammatory immune reaction in healthy participants. In case of hyper-inflammatory reactions due to COVID-19 vaccination, treatment with the IL1 receptor antagonist Anakinra was able to restrain the overshooting immune response to the vaccination [50]. COVID-19 vaccination has been associated with adverse reactions in response to certain treatments, e.g., dermal fillers, like hyaluronic acid, polymethyl-methacrylate and fluid silicone, in the facial region [52]. In this case study, 20 patients, who were injected with dermal fillers, reported swelling and redness as well as other inflammatory hyper-responsiveness at the side of dermal filler injection immediately after a COVID-19 vaccination. It can be hypothesized that the usage of dermal fillers sensitized the body for Polyethylene glycol (PEG) particles [53], which are used as stabilizers in mRNA COVID-19 vaccines and connected to anaphylaxis [54,55]. As PEG is used widely by pharmaceutical, cosmetics and food industries, allergic reactions are a potential source of concern [56,57] and might explain the adverse events after COVID-19 vaccination [58]. Another potential source for inflammatory immune reactions in response to an inoculation with mRNA-based vaccines might be lipid compounds, which are used to encapsulate the mRNA into vesicle for transfection of human cells. A recent study presented evidence for membrane-destabilizing lipids leading to inflammasome activation and induction of pro-inflammatory cytokine secretion in murine bone marrow-derived macrophages and HEK cells [59].

Interestingly, we observed that patients with a critical disease course, who survived the infection (Figure 1F), showed significantly elevated levels of IL10 in their blood. Similar observations have been made by other researchers, who hypothesized that the elevated IL10 levels contribute to the pathogenesis of severe COVID-19 diseases by amplifying viral sepsis-related hyper-inflammation and induction of cytotoxic CD8^+^ T cells [60]. Except for the herein presented data, so far, there have not been published any studies to our knowledge that discriminate the critically ill COVID-19 patients into survived and deceased. Many different immune cells produce the immune suppressive cytokine IL10 contributing to the re-establishment of immune homeostasis after an infection [61]. While the present study did not establish the source of IL10 and limited flow cytometric analysis of the B cell compartment, we speculate that a specialized but heterogeneous subpopulation of B cells, called regulatory B cells (Bregs) contribute to the elevated IL10 levels in critical COVID-19 patients. Breg cells are able to produce IL10 and can be characterized by CD19^+^CD24^hi^CD38^hi^CD1d^hi^ and CD19^+^CD24^hi^CD27^+^ [62].

One aspect of our study was to investigate the dynamics and expansion of class-switched memory B cells and circulating Tfh cells, which are central to the establishment of immunity against pathogens [63]. Similar to Hartley et al. [64], we observed that IgD^−^CD27^+^CD38^low^ class-switch B cells expanded rapidly after the first symptoms appeared and moreover, correlated to increasing α-Spike-Abs titers and numbers of Tfh cells during the initial stage of infection (Figure 1B–D). At later stages of uncleared SARS-CoV-2 infections, we observed higher frequencies of class-switched memory B cells and Tfh cells in deceased critically ill patients in comparison to survivors (Figure 1H,I), leading to an increased Tfh/Treg ratio (Figure 1J). An increase in Tfh/Treg ration has been previously associated with autoimmune diseases, like systemic lupus erythematosus [7]. One might speculate that the elevated Tfh/Treg ratio together with the presence of increased levels of IL10 are signs of the unrestrained immune reactions in severe COVID-19 disease courses which resemble ongoing autoimmune diseases.

The aim of our study was to understand the dynamics of Ab development against SARS-CoV-2 in different vaccination cohorts. These cohorts represent real-life scenarios. Given the fact that even today, infection-preventing Ab titers are not known, we went on to compare resulting Ab titers between different vaccination schemes. In addition, we monitored cytokines as potential inflammatory vaccine response. Despite the limited number of participants, we were able to describe a brought picture of several vaccination strategies and could demonstrate that even cured COVID-19 patients benefit from vaccination. In regard to potential side effects, we could demonstrate that COVID-19 vaccination led to a low-grade, barely detectable systemic inflammatory response, which was in a timely manner restrained and reverted. Through the present study, we hope to further encourage people all over the world to participate in vaccination campaigns to fight the still ongoing pandemic.

## 4. Material and Methods

### 4.1. Study Design and Patient Cohorts

Infection with SARS-CoV-2 was confirmed in all patients with qRT-PCR test or antigen-tests for SARS-CoV-2 in nasopharyngeal swabs, and quantification of SARS-CoV-2 N-protein concentrations and serum-anti-N Ab titers. Characterization of the virus strains was not performed. Samples from mild COVID-19 cases (collected from January to March 2020) and critically ill COVID-19 patients (collected from November 2020 to February 2021) were collected in Germany in the region of North-Rhine-Westphalia. This study was performed in line with the principles of the Declaration of Helsinki. Blood sampling of SARS-CoV-2 infected or recovered patients and healthy controls was approved by the local institutional research ethics board (University Hospital Bonn, ethics vote 468/20). We collected comprehensive clinical and demographic information, medical history, comorbidities and vaccination schedules for all patients and participants.

We categorized patients in the mild cohort when their symptoms included fever, loss of smell and taste, headache and diarrhea, and when they recovered at their private residencies. The quarantine regulations required the help of the Medical Corps of the German Armed Forces to obtain samples of mildly infected patients in their households up to 6 times during the 21 days from inclusion into the study.

Patients with acute respiratory distress syndrome (ARDS) caused by SARS-CoV-2 infection were diagnosed according to the Berlin Definition [65] and categorized as “Critical”. They were included in the study upon admission to the ICU. These patients were also sampled up to six times during the 21 days from inclusion in the study. Treatment included invasive mechanical ventilation or extracorporeal membrane oxygenation (ECMO).

Patients with a history of SARS-CoV-2 infection clinically categorized as cured yet suffering from persisting COVID-19 symptoms at least eight weeks and up to 12 months after infection onset were classified as “Long-COVID”. Patients were recruited at the Long-COVID ambulance of the University Hospital Bonn (Germany) and through Long-COVID support groups on social media (Twitter and Facebook) (UK). Symptoms included fatigue, reduced resilience, cognitive dysfunction, headache, PoTS, tachycardia, palpitations, chest pain and shortness of breath.

Vaccinated volunteers were sampled at various timepoints before and after administration of the vaccine shots (BNT162b2, Sikevax mRNA-1273, Vaxzevria or Janssen) and followed up to one year after the first shot. Participants who did not undergo an active SARS-CoV-2 infection and without any history of prior COVID-19 were categorized as “Control” (Ctrl). Healthy controls were sampled up to 5 times within a fortnight.

### 4.2. Sample Generation and Storage

We used 7.5 mL Z-Gel S-Monovettes (Sarstedt, Nümbrecht, Germany) for peripheral blood. For flow-cytometry, 9 mL of blood was collected in K3E S-Monovettes (Sarstedt). Serum gel tubes were centrifuged at RT for 10 min at 2500× *g*. The cell-depleted serum fraction was transferred to sterile, barcoded polypropylene tubes (Azenta, Chelmsford, MA, USA) and frozen at −80 °C until needed.

### 4.3. Quantification of SARS-CoV-2 N-Protein, α-Spike-Antibodies Titers and Neutralization

SARS-CoV-2 N-protein concentrations in sera were quantified using the S-PLEX SARS-CoV-2 N Kit (Meso Scale Diagnostics, Rockville, MD, USA) according to the manufacturer’s protocol. Similarly, the concentrations of α-SARS-CoV-2 IgG antibodies and their neutralization capacity in sera were quantified using the SARS-CoV-2 Plate 7 (Meso Scale Diagnostics). Immune assays from Meso Scale were acquired via the MESO QuickPlex SQ 120 imager (Meso Scale Diagnostics) and analyzed with the MSD Discovery Workbench (Meso Scale Diagnostics).

### 4.4. Quantification of Cytokines and Markers for Neuroinflammation

We screened serum samples at chosen time points of the SARS-CoV-2 disease course for the expression of pro- and anti-inflammatory cytokines using the Simoa CorPlex Human Cytokine 10-plex Panel 1 assay (Quanterix, Billerica, MA, USA). We targeted the following analytes: IFNγ, IL1β, IL4, IL5, IL6, IL8, IL10, IL12p70, IL22 and TNFα. In addition, markers for neuroinflammation were quantified using the Simoa Human Neurology 4-Plex E assay (Quanterix) for Abeta 40 (Aβ40), Abeta 42 (Aβ42), Glial Fibrillary Acidic Protein (GFAP™) and Neurofilament light (Nf-L). Data for the 10 CorPlex were acquired and analyzed on the SP-X Imaging and Analysis System™ (Quanterix), whereas the Neurology 4-Plex E assay was analyzed on the Simoa^®^ HD-X Analyzer™ (Quanterix). Both kits were used according to the manufacturer’s instructions.

### 4.5. Quantification of B Cell Subtypes and T Follicular Helper Cells by Flow Cytometry

EDTA blood samples were centrifuged at 1200× *g* for 10 min, and the cell pellet was washed once with PBS and treated with ACK lysis buffer (Thermo Fisher, Waltham, MA, USA) for 5 min. The ACK reaction was stopped with an additional wash. Cells were subsequently resuspended in PBS. Up to 2 × 10^6^ live PBMC (per sample) were stained. Viable and dead cells were discriminated by staining with the following Ab compositions for 30 min at 4 °C: LIVE/DEAD™ Fixable Far Red Dead Cell Stain, FITC-conjugated anti-CD45 (HI30), eFluor™ 506-conjugated anti-CD19 (HIB19), PerCP-eFluor™ 710-conjugated anti-IgD (IA6-2), eFluor™ 450-conjugated anti-IgM (SA-DA4), PE-conjugated anti-CD38 (HB7), PE-eFluor610-conjugated anti-CD27 or Super Bright™-conjugated anti-CD24 (eBioSN3), PerCP-conjugated anti-CD4 (SK3), PE-Cyanine7-conjugated anti-CD25 (CD25-4E3) and Alexa Fluor™ 660-conjugated anti-CD127 (eBioRDR5), all from Invitrogen, FITC-conjugated anti-CD3 (OKT3), Brilliant Violet™ 650-conjugated anti-CD4 (RPA-T4), Brilliant Violet™ 421-conjugated anti-CXCR5 (J252D4) and FITC-conjugated anti-CD3 (OKT3) from Biolegend. After surface staining, cells were washed and fixed in 4% PFA for 10 min at room temperature (RT). Single-cell suspensions were acquired on Attune Next Generation (ThermoFishers) and analyzed with FlowJo (version 10.0.7, Tree star).

### 4.6. Software and Tools for Statistical Analysis

Statistical analysis was performed in R (v4.1.2; [66] by the rstatix (v.0.7.0; [67]). Tables were created using the gtsummary package (v1.5.2; [68]). Figures were created with the ggplot package (v3.3.5; [69] and ggpubr (v0.4.0; [70]. The Kruskal–Wallis and Mann–Whitney U tests were used to calculate *p* values. Adjusted *p* values less than 0.05 were considered statistically significant. Deviations are outlined in figure legends.

## Data Availability

Data presented in this manuscript will be made available upon request.

## Supplementary Materials

The supporting information can be downloaded at: https://www.mdpi.com/article/10.3390/ijms232012231/s1.
