# Supplementary material for: Comparative Analysis of Antibody Titers against the Spike Protein of SARS-CoV-2 Variants in Infected Patient Cohorts and Diverse Vaccination Regimes
Source: Int J Mol Sci. 2022 Oct 13;23(20):12231. doi: 10.3390/ijms232012231 (PMC9602709; doi:10.3390/ijms232012231)
Supplement: Supplementary file 1 [file ijms-23-12231-s001.zip › ijms-1931459-supplementary.pdf]

## Figure S1

**A**

| **Characteristic** | **Ctrl**, N = 17[1] | **Mild**, N = 15[1] | **Critical**, N = 17[1] |
|---|---|---|---|
| **Age** | 57 (49, 62) | 54 (34, 73) | 63 (53, 66) |
| **Sex** | | | |
| f | 7 (41%) | 9 (60%) | 2 (12%) |
| m | 10 (59%) | 6 (40%) | 15 (88%) |
| **Overweight** | 3 (18%) | 1 (7.1%) | 1 (5.9%) |
| Unknown | 0 | 1 | 0 |
| **Hypertension** | 4 (24%) | 3 (21%) | 7 (41%) |
| Unknown | 0 | 1 | 0 |
| **Diabetes** | 2 (12%) | 1 (7.1%) | 2 (12%) |
| Unknown | 0 | 1 | 0 |

[1] Median (IQR); n (%)

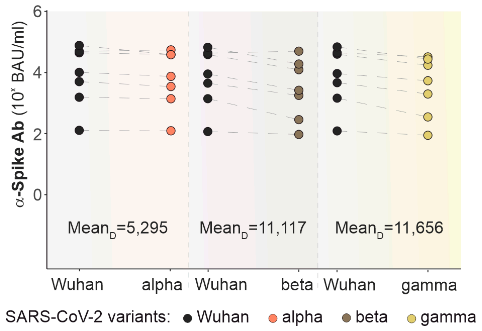


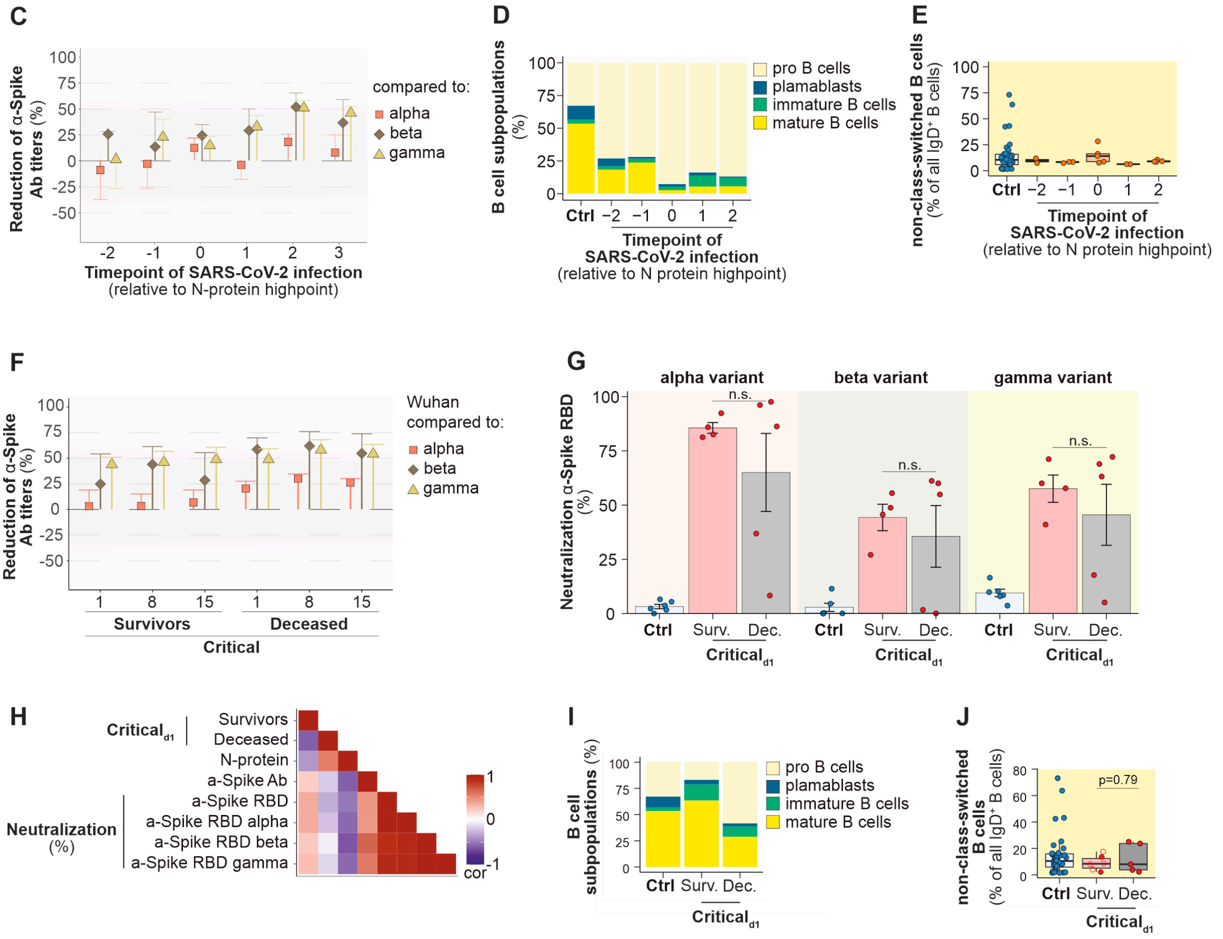


**Figure S1:** ***Dynamics of Ab titers against SARS-CoV-2 variants in different COVID-19 disease stages.*** **(A)** Table with patient data and comorbidities for SARS-CoV-2 infected cohorts and controls. **(B)** Dotplot for specific α-Spike-Ab concentrations against SARS-CoV-2 variants at the timepoint of highest N-protein concentrations ($t_2$) in sera of patients with mild SARS-CoV-2 infection. **(C, F)** Lollypop plot for the percentage point reduction of α-Spike-Ab concentrations against SARS-CoV-2 variants when compared to the Wuhan-variant in mild (C) and critical (E) COVID-19 cases, as well as for (H) Long-COVID patients. **(D, E, I, J)** Frequencies of immune cells in mild (D, E) and critical (G, H) COVID-19 cases, for B cell populations (D, G) and non-class-switched B cells (G, H). **(G)** Neutralization capacity of antibodies against Spike-RBD of the alpha, beta and gamma variants of SARS-CoV-2 in healthy non-vaccinated controls and critically ill patients. **(H)** Pearson correlation matrix of selected parameters including neutralization capacity of α-Spike-Ab for RBD in patients with critical disease course at day 1 of hospitalization. Blue line, mean concentration in healthy Ctrl; blue dashed line, 95% confidence interval; Error bars in (C) and (F) indicate standard error of the mean; Mann-Whitney U test; unadjusted p-value (H); *pVal<0.05, **pVal<0.01, ***pVal<0.001

## Figure S2

### A

| Characteristic | LC control, N = 8[1] | Long COVID, N = 20[1] |
|---|---|---|
| **Age** | 40 (34, 46) | 39 (30, 47) |
| **Sex** | | |
| f | 6 (75%) | 12 (60%) |
| m | 2 (25%) | 8 (40%) |
| **Overweight** | 0 (0%) | 3 (23%) |
| Unknown | 3 | 7 |
| **Hypertension** | 1 (12%) | 2 (10%) |
| Unknown | 0 | 0 |
| **Diabetes** | 0 (0%) | 1 (5.0%) |
| Unknown | 0 | 0 |

[1] Median (IQR); n (%)

### B

| Characteristic | LC control, N = 8[1] | Long-COVID, N = 20[1] | p-value[2] |
|---|---|---|---|
| **Fatigue** | 7 (88%) | 16 (80%) | >0.9 |
| **Cardiovascular System** | 5 (62%) | 10 (50%) | 0.7 |
| **Neurological** | 6 (75%) | 16 (80%) | >0.9 |
| **Insomnia** | 2 (25%) | 3 (15%) | 0.6 |
| **Headaches** | 3 (38%) | 6 (30%) | >0.9 |
| **Dyspnea** | 4 (50%) | 11 (55%) | >0.9 |

[1] n (%)
[2] Fisher's exact test

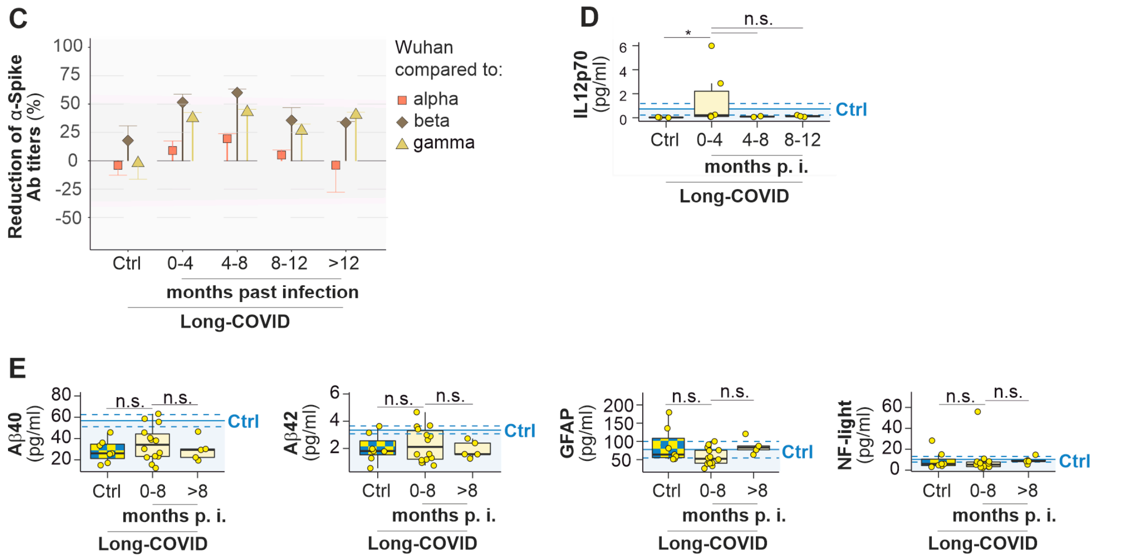


**Figure S2:** ***Dynamics of Ab titers against SARS-CoV-2 variants in Long-COVID.*** **(A)** Table on clinical data of Long-COVID and respective control patients. **(B)** Table on frequencies of symptoms of Long-COVID and LC controls cohorts. (C) Relative α-Spike-Ab titers against SARS-CoV-2 variants in Long-COVID patients. **(D)** Longitudinal analysis of IL12p70 concentration in sera of patients with Long-COVID symptoms and respective controls. **(E)** Longitudinal analysis for concentrations of markers for neuroinflammation Aβ40 (far left), Aβ42 (left), GFAP (right), NFlight (far right) in sera of patients with Long-COVID and LC controls. Blue line, mean concentration in healthy Ctrl; blue dashed line, 95% confidence interval; Error bars in (D) and (E) indicate standard error of the mean; Mann-Whitney U test; unadjusted p-value (D, E); *pVal<0.05, **pVal<0.01, ***pVal<0.001

## Figure S3

### A

| Characteristic | **Impf. Astra**, N = 16[1] | **Impf. Biontech**, N = 45[1] | **Impf. Moderna**, N = 15[1] | **p-value**[2] |
|---|---|---|---|---|
| **Age** | 50 (44, 54) | 47 (37, 54) | 27 (22, 36) | <0.001 |
| **Sex** | | | | 0.013 |
| f | 15 (94%) | 31 (69%) | 7 (47%) | |
| m | 1 (6.2%) | 14 (31%) | 8 (53%) | |

[1] Median (IQR); n (%)
[2] Kruskal-Wallis rank sum test; Fisher's exact test

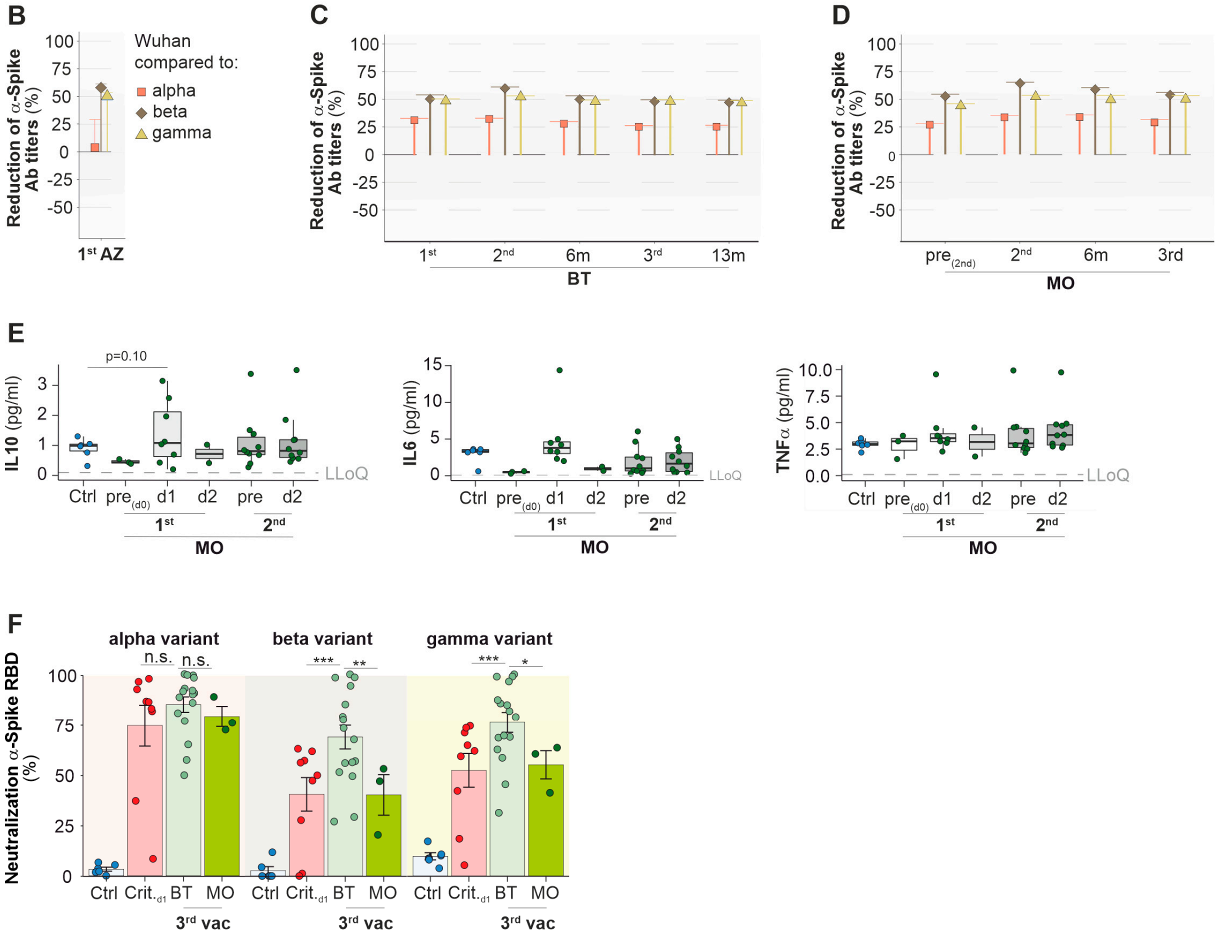


**Figure S3:** ***Dynamics of Ab titers against SARS-CoV-2 variants in different vaccination cohorts.*** **(A)** Table on age and gender for participants vaccinated with AZ, BT and MO. **(B-D)** Lollypop plot for the percentage point reduction of α-Spike-Ab concentrations against SARS-CoV-2 variants when compared to the Wuhan-variant in participants vaccinated with AZ (B), BT (C) and MO (D) at indicated timepoints of the vaccination schedule. **(E)** Concentrations of cytokine concentrations for IL10 (left), IL6 (middle), and TNFα (right) in peripheral blood of participants vaccinated with MO before and after inoculation. **(F)** Neutralization capacity of α-Spike-Ab against RBD of SARS-CoV-2 alpha, beta and gamma variants in healthy non-vaccinated controls, critically ill COVID-19 patients, and vaccinated individuals with BT and MO six months after second booster ($3^{rd}$ vac). Error bars in (C), (F) and (J) indicate standard error of the mean; Mann-Whitney U test; unadjusted p-value (E); *pVal<0.05, **pVal<0.01, ***pVal<0.001

## Figure S4

### A

| Characteristic | Astra/Astra/Biontech N = 3[1] | Astra/Biontech/Biontech N = 4[1] | J&J/Biontech N = 3[1] | Moderna/Moderna/Biontech N = 5[1] | Astra/Biontech N = 20[1] | p-value[2] |
|---|---|---|---|---|---|---|
| **Age** | 60 (44, 61) | 43 (30, 56) | 35 (32, 42) | 36 (22, 60) | 32 (27, 46) | 0.5 |
| **Sex** | | | | | | 0.004 |
| f | 0 (0%) | 4 (100%) | 0 (0%) | 2 (40%) | 14 (70%) | |
| m | 3 (100%) | 0 (0%) | 3 (100%) | 3 (60%) | 6 (30%) | |

[1] Median (IQR); n (%)
[2] Kruskal-Wallis rank sum test; Fisher's exact test

### B

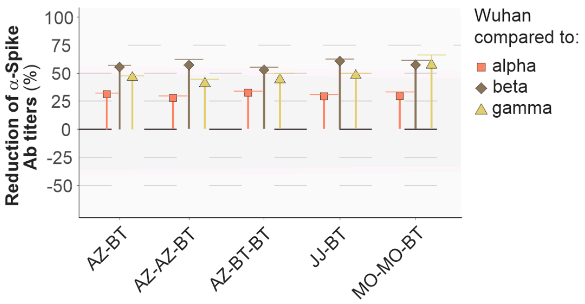


**Figure S4:** ***Dynamics of Ab titers against SARS-CoV-2 variants multiple vaccines in cross-over vaccination schemes.*** **(A)** Table on age and gender for participants vaccinated multiple times with AZ, JJ, BT and MO. **(B)** Comparative lollypop plot for the percentage point reduction of α-Spike-Ab concentrations against SARS-CoV-2 variants, after the vaccination with multiple vaccines in diverse vaccination schemes. Error bars show standard error of the mean

# Figure S5

## A

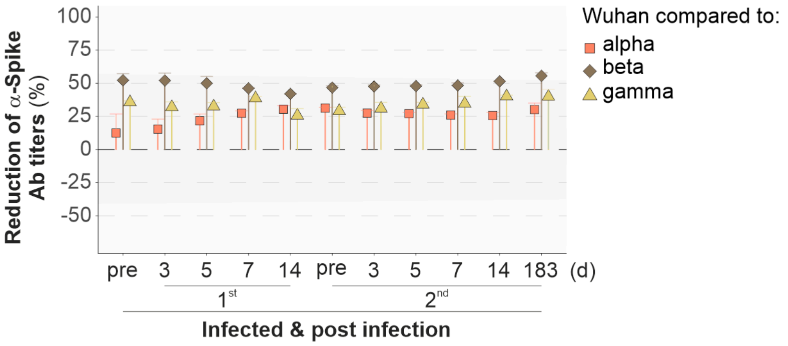


**Figure S5:** ***Ab titers against the different variant compared to Wuhan Sars-CoV-2 Spike protein for the cohort of previously infected and subsequently vaccinated patients.*** **(A)** Lollypop plot showing reduction of Ab titers compared to Wuhan variant for the indicated variants. Error bars indicated standard error of the mean. N = 3
